# NFAT5 and HIF-1α Coordinate to Regulate NKCC1 Expression in Hippocampal Neurons After Hypoxia-Ischemia

**DOI:** 10.3389/fcell.2019.00339

**Published:** 2019-12-13

**Authors:** Xing-Liang Yang, Meng-Liu Zeng, Lin Shao, Guang-Tong Jiang, Jing-Jing Cheng, Tao-Xiang Chen, Song Han, Jun Yin, Wan-Hong Liu, Xiao-Hua He, Bi-Wen Peng

**Affiliations:** ^1^Department of Physiology, Hubei Provincial Key Laboratory of Developmentally Originated Disease, School of Basic Medical Sciences, Wuhan University, Wuhan, China; ^2^Department of Pathophysiology, School of Basic Medical Sciences, Wuhan University, Wuhan, China; ^3^Department of Immunology, School of Basic Medical Sciences, Wuhan University, Wuhan, China

**Keywords:** HI, neurons, NKCC1, NFAT5, HIF-1α

## Abstract

Hypoxic-ischemic encephalopathy (HIE) is a serious birth complication with severe long-term sequelae such as cerebral palsy, epilepsy and cognitive disabilities. Na^+^-K^+^-2Cl^–^ cotransporters 1 (NKCC1) is dramatically upregulated after hypoxia-ischemia (HI), which aggravates brain edema and brain damage. Clinically, an NKCC1-specific inhibitor, bumetanide, is used to treat diseases related to aberrant NKCC1 expression, but the underlying mechanism of aberrant NKCC1 expression has rarely been studied in HIE. In this study, the cooperative effect of hypoxia-inducible factor-1α (HIF-1α) and nuclear factor of activated T cells 5 (NFAT5) on NKCC1 expression was explored in hippocampal neurons under hypoxic conditions. HI increased HIF-1α nuclear localization and transcriptional activity, and pharmacological inhibition of the HIF-1α transcription activity or mutation of hypoxia responsive element (HRE) motifs recovered the hypoxia-induced aberrant expression and promoter activity of NKCC1. In contrast, oxygen–glucose deprivation (OGD)-induced downregulation of NFAT5 expression was reversed by treating with hypertonic saline, which ameliorated aberrant NKCC1 expression. More importantly, knocking down NFAT5 or mutation of the tonicity enhancer element (TonE) stimulated NKCC1 expression and promoter activity under normal physiological conditions. The positive regulation of NKCC1 by HIF-1α and the negative regulation of NKCC1 by NFAT5 may serve to maintain NKCC1 expression levels, which may shed light on the transcription regulation of NKCC1 in hippocampal neurons after hypoxia.

## Introduction

Newborns with hypoxic-ischemic encephalopathy (HIE) usually have loss of hippocampal neurons (Arriaga-Redondo et al., 2019) and aberrant hippocampal neurogenesis (Mattiesen et al., 2009; Hu et al., 2017). Two phases of neuronal death in HIE have been identified in both clinical and experimental studies (Penrice et al., 1996; Dixon et al., 2015). First, exhaustion of the cell’s energy stores induces immediate neuronal death and, second, delayed neuronal death occurs after a latent period of at least 6 h, which is associated with encephalopathy and increased seizure activity.

NKCC1 (encoded by *SLC12A2*) belongs to the subfamily of cation-chloride cotransporters that are involved in cell volume and intracellular Cl^–^ concentration ([Cl^–^]_*i*_) regulation (Watanabe and Fukuda, 2015). NKCC1 mRNA expression level is high in the early postnatal period but decreases during postnatal development (Wang et al., 2002). NKCC1 is an intrinsic membrane protein that transports chloride ions, together with sodium and/or potassium ions, across the plasma membranes of cells. The activity of NKCC1 determines the [Cl^–^]_*i*_. Neurons, glia, endothelial cells and epithelial cells that line the brain’s ventricular system regulate [Cl^–^]_*i*_ to help maintain their cellular volume amidst changes of extracellular osmolality and intracellular solute content (Simard et al., 2010). Bumetanide, an NKCC1-specific inhibitor, is used to treat aberrant NKCC1 expression related diseases (Kahle and Staley, 2008; Kharod et al., 2019).

As regulators of gene expression programs, transcription factors exert key functions to control and maintain the function of hippocampal neurons (Beckervordersandforth et al., 2015; Leal et al., 2017). Hypoxia-inducible factor-1 (HIF-1) is a transcription factor that consists of α and β subunits and its target genes contain hypoxia responsive element (HRE) motifs (5′-(A/G)CGTG-3′) (Huang, 2013). HIF-1α is commonly associated with hypoxia-dependent tissue edema (Martin, 2001) by regulating ion and water transporters such as NKCC1 (Ibla et al., 2006; Lu et al., 2015), cystic fibrosis transmembrane regulator (CFTR) (Zheng et al., 2009) and aquaporin (AQP) (Mou et al., 2010; Johnson et al., 2015). In the central nervous system, HIF-1α is stabilized by insults associated with hypoxia and ischemia (Vangeison et al., 2008). Because most of its target genes mediate both adaptive and pathological processes (Ratan et al., 2004; Sheldon et al., 2009; Barteczek et al., 2017), the role of HIF-1α in neuronal survival is debated.

NFAT5, also known as tonicity-responsive enhancer binding protein (TonEBP), can maintain cellular homeostasis by regulating various osmoprotective-related genes under physiological conditions (Yang et al., 2018). NFAT5 was recently characterized as a hypoxia-inducible protein (Dobierzewska et al., 2015) and its target genes contain tonicity enhancer element (TonE) [5′-TGGAAA(C/A/T)A(T/A)-3′] (Lopez-Rodriguez et al., 2001). NFAT5 activation is increased after hypertonic saline (HS) stimulation (Kojima et al., 2010) and HS alleviates cerebral edema by inhibiting NKCC1 upregulation (Huang et al., 2014). In the central nervous system, NFAT5 is highly enriched in the nuclei of neurons (Maallem et al., 2006) but its role in neurons has barely been explored.

NKCC1 is significantly upregulated after hypoxia-ischemia (HI), which aggravates brain edema, aberrant hippocampus neurogenesis and blood-brain barrier (BBB) disruption (Hu et al., 2017; Luo et al., 2018). The consequences of abnormal NKCC1 expression in HIE have been well explored, but the transcriptional regulation of its expression is not fully understood. Here, we show that NKCC1 is significantly upregulated in hippocampal neurons after hypoxia, which increases [Cl^–^]_*i*_. The transcription factors HIF-1α and NFAT5 cooperatively govern these activities. The upregulation of NKCC1 by HIF-1α and downregulation of NKCC1 by NFAT5 may serve to maintain the NKCC1 level, which sheds light on the transcriptional regulation of NKCC1 in hippocampal neurons.

## Materials and Methods

### Animal Models and Treatments

Sprague-Dawley (SD) rat were provided by the Hubei Province Center for Animal Experiments. Animal experiments were approved by the Care and Use Committee of Wuhan University Medical School. The rats kept in a room maintained at 25 ± 2°C and a relative humidity of 60–80% with a 12 h light-dark cycle. All rats were housed at the animal facility of the animal biosafety level III (ABSL-III) laboratory of Wuhan University. The day of the birth was defined as postnatal day 0 (P0). P7 rat pups (*n* = 180) randomly divided into the six groups (*n* = 30 each group): Sham, HI (3 h), HI (6 h), HI (12 h), and HI (24 h).

### Neonatal HI Model

A well-characterized model of neonatal HI was prepared as previously described (Vannucci and Vannucci, 1997). P7 rats of both genders (body weight 15 ± 1 g, equal number of males and females in each group) were anesthetized by inhalation of isoflurane. Sterilized skin was incised with ophthalmology scissors. The *right* pulsating carotid artery was then carefully separated. The upper and lower ends of the *right* carotid artery were tied using 4-0 surgical sutures before cutting the artery in the middle. The skin incision was sutured with the same surgical suture. All surgical instruments were sterilized. After 2 h of recovery, the pups were placed in an airtight transparent chamber, and the chamber was placed into a 37°C incubator to maintain a constant thermal environment. The pups were maintained in 8% O_2_ in N_2_ for 2.5 h. After the hypoxic process, the pups were put back in the cages. Each successful HI model showed significant edema in the ipsilateral hemisphere. The sham group, which underwent anesthesia with neck incision and suture, did not exhibit this edema. The mortality of the model rats was about 10%.

### Cell Culture and Plasmids

The hippocampal neurons from the P0 rats were prepared and cultured as previously described (Kaech and Banker, 2006). P0 mice were euthanized after being disinfected with 75% ethanol. Brain tissue was isolated and then placed in pre-cooled phosphate-buffered saline (PBS). To obtain dissociated cells, the meninges were removed and the clean hippocampus was digested in Hank’s balanced salt solution (HBSS) containing 0.125% trypsin at 37°C for 10 min. Complete growth medium [neurobasal medium, 2% B27(Gibco), 1% L-glutamine and 1% penicillin/streptomycin] was added to terminate the digestion. The digested tissues were gently mixed with a pipette, and then centrifuged at 70 *g* for 5 min. Dissociated cells were seeded in plates and then maintained in a conventional cell culture incubator (37°C, 5% CO_2_) for 10–12 days.

Rat adrenal medulla chromaffin tumor cell lines (PC12) were obtained from Shanghai Cell Research Center (Shanghai, China). These cells were grown and maintained in Roswell Park Memorial Institute (RPMI) 1640 supplemented with 10% fetal bovine serum and 1% 100U/ml penicillin/streptomycin. In a humidified 5% CO_2_/95% air atmosphere at 37°C, the cells were plated in a 75 cm^2^ cell culture flask (Corning, Acton, MA, United States) and were split twice a week. For the experiments, the cells were plated on 24-well dishes (2–4 × 10^5^cells/well).

A luciferase reporter plasmid with an NKCC1 promoter (∼2 kb), pGL3-NKCC1p, was constructed by cloning the relevant fragments into the pGL3-Basic vector in frame at the 5′ *Xho*I and 3′ *Hin*dIII sites and was transfected in PC12. The primer lists are shown in Supplementary Table S1. In addition, NFAT5 and HIF-1α binding sites of the NKCC1 promoter were mutated with various point mutations and corresponding plasmids were then constructed (Figure 5E).

### Oxygen–Glucose Deprivation (OGD)

Oxygen–glucose deprivation was conducted as described previously (Luo et al., 2018). Briefly, the hippocampal neurons and PC12 were grown in complete growth media as monolayers in a cell culture incubator (95% O_2_ and 5% CO_2_ at 37°C). To initiate OGD *in vitro*, the plates were washed with PBS three times. For the OGD group, OGD medium (serum- and glucose-free Dulbecco’s Modified Eagle Medium [DMEM]) was added. For the HS group, OGD medium + HS (serum- and glucose-free DMEM + 100 mM NaCl) was added. For the KC7F2 (C_16_H_16_Cl_4_N_2_O_4_S_4_) group, OGD medium + KC7F2 [serum- and glucose-free DMEM + 10 mM of the inhibitor of HIF-1α pathway, KC7F2 (T3169, TargetMol, Shanghai, China)] was added. The cells were then placed in a hypoxic/anoxic chamber (1% O_2_, 5% CO_2_, and 94% N_2_ at 37°C). After 3.5 h, the plates were removed from the anaerobic chamber and the medium was changed to complete growth medium. For the glucose-containing control group, these cells were kept in a regular incubator (5% CO_2_ and 95% O_2_) and the medium was changed to fresh complete growth medium at the same time as in the OGD group. Cell extracts were collected after OGD for the following experiments.

### Immunofluorescence Staining

Immunofluorescence staining was carried out using antibody against NKCC1 (Santa Cruz Biotechnology, CA, United States; used at 1:50), NFAT5 (Abcam, Cambridge, MA, United States; used at 1:100) and HIF-1α (Abcam, Cambridge, MA, United States; used at 1:100) in peri-ischemic hippocampal brain slices and in primary cultured hippocampal neurons. Microtubule-associated protein 2 (MAP2, ABclonal, Wuhan, China; used at 1:200) was used as the neuronal mark. The protocol is detailed in the Supplementary Material.

### Quantification of Immunofluorescence Intensity and Image Analysis

For counting NKCC1 positive cell in ipsilateral hemisphere, 5 brain slices of each rat were analyses in histological analysis as previously described (Tozaki-Saitoh et al., 2019). The NKCC1 positive cell counts were performed using a Pannoramic Digital Slide Scanners (3D HISTECH, Ltd.) in the brain cortex. These data are showed as mean value of cells/slices, based on average amount of cells in 5 slices.

For quantitative assessment of the immunofluorescence intensity of NKCC1, hif-1α and NFAT5 in hippocampal neurons, 10 fields/well were analyses and divided by the number of cells counterstained with DAPI. The pixel intensities within each field were measured by ImageJ^1^ (NIH, Bethesda, MD, United States). The average value was normalized to that of the control.

### Quantitative PCR (qPCR)

Total RNA was extracted from the ischemic cerebral cortices and the hippocampus-derived neurons of SD rats in the different groups using TRIzol reagent (Invitrogen Life Technologies Corporation, Carlsbad, CA, United States) to detect the mRNA levels of NKCC1, NFAT5 and HIF-1α. The protocol is detailed in the Supplementary Material.

### Measurement of Intracellular Cl^–^ Concentration ([Cl^–^]_*i*_)

*N*-(ethoxycarbonylmethyl)-6-methoxyquinolinium bromide (MQAE, Invitrogen, Cambridge, MA, United States), a chloride-sensitive fluorescent indicator inversely related to intracellular chloride ion concentration, was used to detect [Cl^–^]_*i*_. This dye detects the ion via diffusion-limited collisional quenching. The cultured hippocampal neurons were incubated with 10 mM MQAE in a Kreb HEPES-buffered isotonic solution (DMEM, 0.1% BSA, 10 mM 4-(2-hydroxyethyl)-1 piperazine-ethanesulfonic acid [HEPES], pH 7.5) for 1 h at 37°C. Subsequently, cells were washed with DMEM three times. Fluorescence was excited every 60 s at 340 nm, and emission fluorescence at 460 nm was recorded. Images were collected and analyzed with the Image-Pro Plus 6.0 image-processing software.

In order to quantitative assess the immunofluorescence intensity of MQAE in primary cultured hippocampal neurons, a total of 50 MQAE positive neurons in each field and 5 fields/well were analyses. The relative quantization of MQAE intensity of each well is represented as the average value of a total of 250 cells in each well. MQAE intensity of each neuron was measured by ImageJ^1^ (NIH, Bethesda, MD, United States).

### Co-immunoprecipitation (Co-IP) Assays and Western Blotting

Co-IP assays and western blotting were performed as described previously (Huang et al., 2015). The hippocampal neurons were lysed in radioimmunoprecipitation assay (RIPA) buffer (50 mM Tris-HCl, pH 7.4, 150 mM NaCl, 0.5% Triton X-100 and 1 mM phenylmethylsulfonyl fluoride [PMSF]) for 15 min. The cells were centrifuged at 12,000 *g* at 4°C for 30 min to remove the cell debris. Five percent of the cell lysates were kept as the input samples, and the remaining lysates were precleared with protein A- or protein G-coupled Sepharose (Life Technologies) for 2 h at 4°C and then immunoprecipitated with the antibodies (against NFAT5, HIF-1α and IgG) for 12 h at 4°C. The immunoprecipitates were washed four times with RIPA buffer and then boiled in sodium dodecyl sulfate (SDS) loading buffer for western blot analysis.

The details of western blotting are provided in the Supplementary Material.

### Chromatin Immunoprecipitation (ChIP)

The ChIP protocol was adapted from described previously (Sun et al., 2014) with some modifications. Neurons were cross-linked in PBS containing 1% formaldehyde for 10 min at room temperature and quenched with 500 μl 2.5 M glycine. After crosslinking, the cells were washed with PBS twice and resuspended in 1 ml lysis buffer (50 mM Tris-HCl, pH 8, 0.5% SDS and 5 mM ethylenediaminetetraacetic acid [EDTA]) for 10 min at 4°C. The lysates were subjected to sonication to obtain 200- to 500-bp fragments of DNA (10 min cycle, 5 s pulses; amplitude, 30%) and then were centrifuged at 12,000 *g* at 4°C for 10 min to obtain the supernatants. Next, 10% of the supernatants were kept as input samples, and the remaining were divided according to the antibodies (against NFAT5, HIF-1α and IgG). Each sample was diluted 1:4 with dilution buffer (20 mM Tris-HCl, pH 8, 150 mM NaCl, 2 mM EDTA and 1% Triton X-100). The samples were precleared with pretreated protein A or G beads (1 mg/ml bovine serum albumin), 1 mg/ml sperm DNA and 20% beads) for 2 hr at 4°C. The aliquots were then incubated overnight at 4°C with pretreated protein A or G beads and antibodies (against NFAT5, HIF-1α and IgG). After extensive washing (four times each) with RIPA buffer, wash buffer (20 mM Tris-HCl, pH 8.0, 1 mM EDTA, 250 mM LiCl, 0.5% NP-40 and 1 mM PMSF) and TE buffer (10 mMTris-HCl, pH8.0 and 1 mMEDTA), the beads were resuspended in TE buffer. The resuspended beads were subjected to RNase A and proteinase K digestion, and the crosslinking was reversed at 65°C for 8–10 h. DNA was recycled with a DNA purification kit (Tiangen, Beijing, China). And ChIP-qPCR data were normalized using the fold enrichment method (ChIP signals were divided by IgG signals).

### Transfection

NFAT5-specific small interfering RNA (siRNA) was designed and synthesized by GenePharma Company (GenePharma, Shanghai, China). The siRNA transfection procedures are described in the manufacturer’s protocol for the transfection kit (Polyplus, Strasbourg, France). The siRNA was diluted in PBS, vortexed 10 s and spun down. Next, jetPRIME reagent was added and the mixture was vortexed 10 s, spun down and incubated for 10 min at room temperature. The siRNA transfection mixture was then added to a plate and the hippocampal neurons were harvested at 36 h after transfection.

The plasmid transfection procedures are described in the manufacturer’s protocol for the transfection kit (Invitrogen). The luciferase reporter plasmid was diluted in Opti-MEM (Gibco) and diluted Lipofectamine 2000 (Invitrogen). The mixture was incubated for 10 min at room temperature. The plasmid transfection mixture was then added to a plate and PC12 were harvested at 36 h after transfection.

### Luciferase Assay

The luciferase assay procedures are described in the manufacture’s protocol for the luciferase assay system (Promega, Madison, WI, United States). The pGL3-basic plasmid, the plasmid expressing NKCC1 promoter and the mutated ones were transfected into PC12. The total amount of DNA was normalized with an empty vector during transfection. PC12 were harvested at 36 h after transfection for the luciferase assay.

### Statistical Analysis

Prism 7 software was used to analyze the data and construct graphs (Including which tests were performed, exact *P*-values, and sample sizes). In brief, one-way analysis of variance (ANOVA) with test for linear trend followed by Tukey’s test were used, as appropriate, to analyze the data. At least three independent experiments were performed. Bias was reduced by making sure that the researchers conducting the data collecting and analysis were blinded. *P* < 0.05 was considered significant. Pearson’s correlation coefficient was used to investigate the relationship between two quantitative. The data are presented as the mean ± SEM.

## Results

### NKCC1 Is Upregulated in the Hippocampus and in Hippocampal Neurons

**FIGURE 1:**
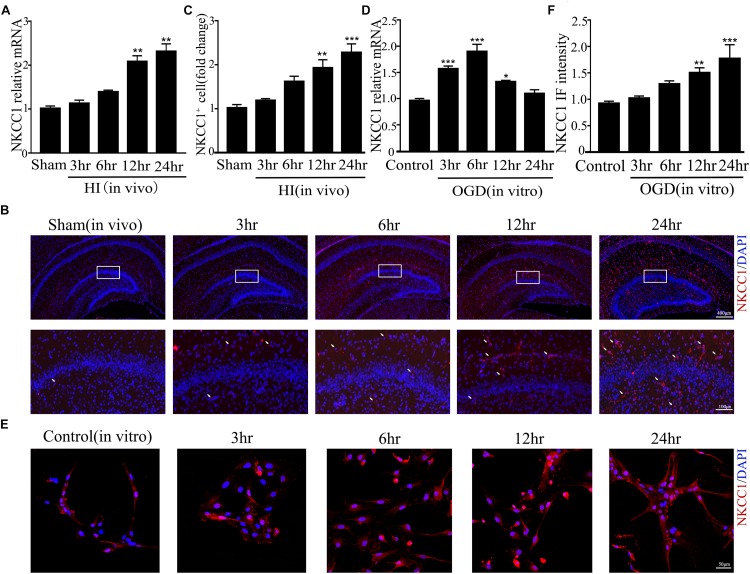
NKCC1 expression after HI and OGD. **(A)** The NKCC1 mRNA expression level at 3, 6, 12, and 24 h after neonatal HI. The mRNA was collected in the ipsilateral hemisphere from sham and HI groups. **(B)** NKCC1-positive cells (indicated by white arrows and counterstained with 4′,6-diamidino-2-phenylindole [DAPI]) were examined in the ipsilateral hemisphere sections at 3, 6, 12, and 24 h, after neonatal HI. **(C)** The relative number of NKCC1-positive cells in the ipsilateral hemisphere hippocampus. **(D)** The NKCC1 mRNA expression level at 3, 6, 12, and 24 h in primary cultured hippocampal neurons, after OGD. **(E)** Confocal image demonstrating NKCC1 expression in neurons. **(F)** The intensity of NKCC1 was determined in 10 fields/well and divided by the number of cells counterstained with DAPI. The values represent the mean ± SEM. ^∗^*p* < 0.05, ^∗∗^*p* < 0.01, ^∗∗∗^*p* < 0.001 versus control (Tukey’s test after one-way ANOVA).

Our previous research showed that NKCC1 was a potential target for HIE therapy, its ab-normal expression induces aberrant hippocampal neurogenesis and BBB leakage (Hu et al., 2017; Luo et al., 2018). However, it was unknown how it was upregulated by hypoxia. Therefore, we verified NKCC1 upregulation timing *in vivo* (hippocampal brain slices) and *in vitro* (hippocampal neurons) after hypoxia. *In vivo*, the NKCC1 mRNA level was gradually upregulated at 12 and 24 h after neonatal HI (Figure 1A). Immunofluorescence was used to determine the NKCC1 expression in coronal brain slices following neonatal HI, which revealed increased NKCC1 positive cells in the hippocampus at 12 and 24 h compared to in the sham group (Figures 1B,C). Primary cultured hippocampal neurons were used to further confirm this phenomenon *in vitro*. NKCC1 mRNA level increased at 3, 6, and 12 h after OGD, peaking at 6hr after OGD (Figure 1D). The fluorescence intensity of NKCC1 gradually increased at 12 and 24 h (Figures 1E,F and Supplementary Figure S1C). Thus, NKCC1 was upregulated after hypoxia, but the changes *in vivo* and *in vitro* were not completely consistent.

### Hypoxia-Induced NKCC1 Upregulation Is HIF-1α Dependent

**FIGURE 2:**
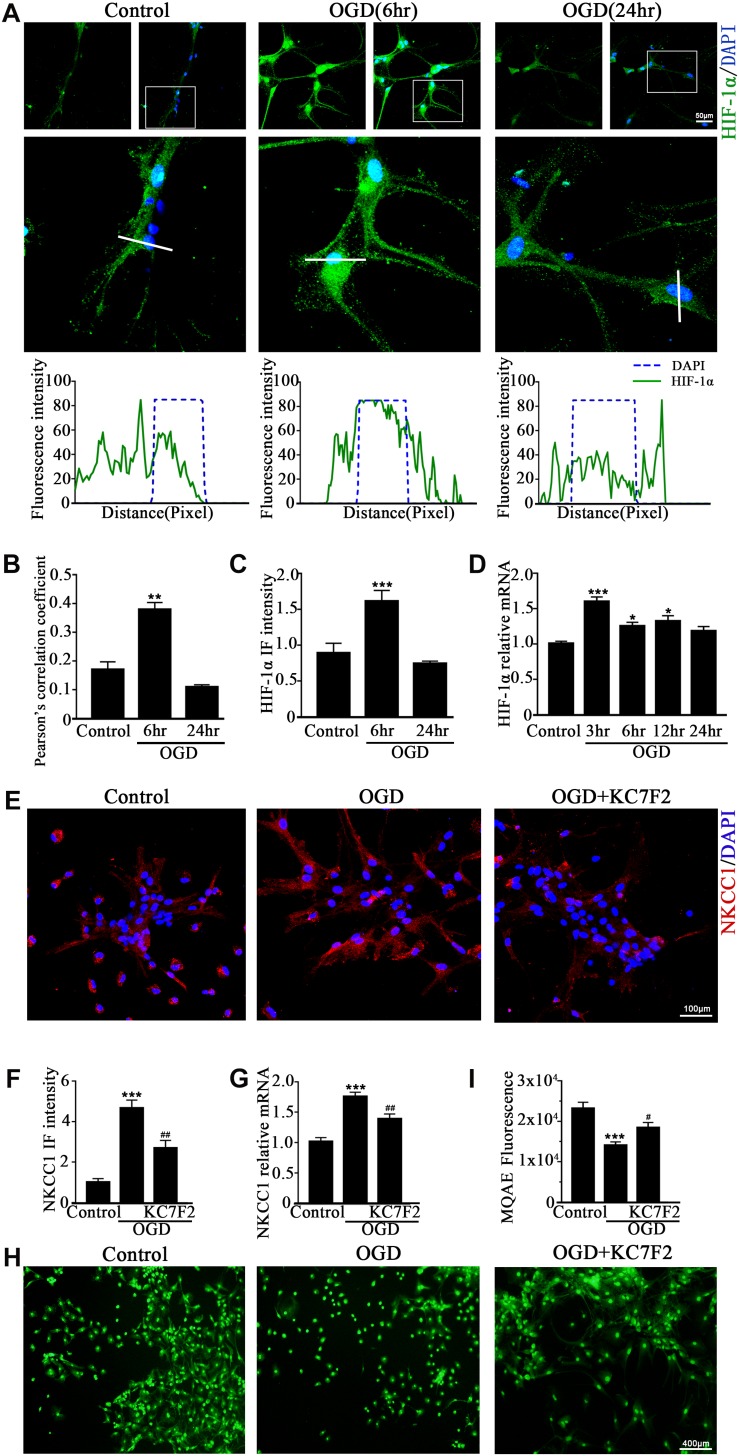
HIF-1α mediates NKCC1 expression. (**A**, top) Confocal images showing co-localization of HIF-1α and DAPI in neurons. (**A**, middle) Enlarged views of indicated co-localization between HIF-1α with DAPI. (**A**, bottom) Side overlap of two peaks indicated partial co-localization. **(B)** Pearson’s correlation coefficient is shown in the graph from the analysis of independent experiments. **(C)** The intensity of HIF-1α fluorescence was determined in 10 fields/well and divided by the number of cells counterstained with DAPI. **(D)** HIF-1α mRNA expression level at 3, 6, 12, and 24 h after OGD. **(E)** Confocal image demonstrating NKCC1 expression in neurons after treatment with KC7F2 (10 mM). **(F)** The intensity of NKCC1 was determined in 10 fields/well and divided by the number of cells counterstained with DAPI. **(G)** NKCC1 mRNA level in neurons after treatment with KC7F2. **(H,I)** Fluorescence imaging of Cl^–^ via MQAE staining in neurons. The green fluorescence indicates the intensity of MQAE. The values represent the mean ± SEM. ^∗^*p* < 0.05, ^∗∗^*p* < 0.01, ^∗∗∗^*p* < 0.001 versus control (Tukey’s test after one-way ANOVA). ^#^*p* < 0.05, ^##^*p* < 0.01 versus OGD (Tukey’s test after one-way ANOVA).

A previous study showed that HIF-1α mediated hypoxia-dependent NKCC1 expression in intestinal epithelial cells (Ibla et al., 2006). Thus, we checked whether HIF-1α could regulate NKCC1 expression in hippocampal neurons. The fluorescence intensity and nuclear localization of HIF-1α (Figures 2A–C and Supplementary Figure S1B) were increased at 6 h (the peak NKCC1 mRNA time point) while they were decreased to the normal levels at 24 h (the saturation NKCC1 protein time point). HIF-1α mRNA increased at 3, 6, and 24 h and peaked at 3 h (Figure 2D). It seems that enhanced NKCC1 transcription accompanied HIF-1α nuclear localization. Chemical inhibition of HIF-1α transcription activation by treatment with 10 μM KC7F2 significantly reduced NKCC1 mRNA (Figure 2G) and the fluorescence intensity (Figures 2E,F). Neuronal [Cl^–^]_*i*_ indicated by MQAE fluorescence intensity was also reduced after KC7F2 treatment (Figures 2I,H). These results suggest that HIF-1α is critical for OGD-induced NKCC1 regulation.

### NFAT5 Is Essential for Moderate Expression of NKCC1 in Neurons

NFAT5 is a transcription factor that sensitive to extracellular osmolarity and its transcription activation is dramatically increased after HS stimulation (Yang et al., 2018). HS, which inhibits NKCC1 expression (Huang et al., 2014; Rasmussen et al., 2015) is an efficient way to treat brain edema in clinical settings (Bhardwaj and Ulatowski, 2004; Wu et al., 2019). Hence, we assumed that NFAT5 mediate NKCC1 expression in hippocampal neurons after OGD.

**FIGURE 3:**
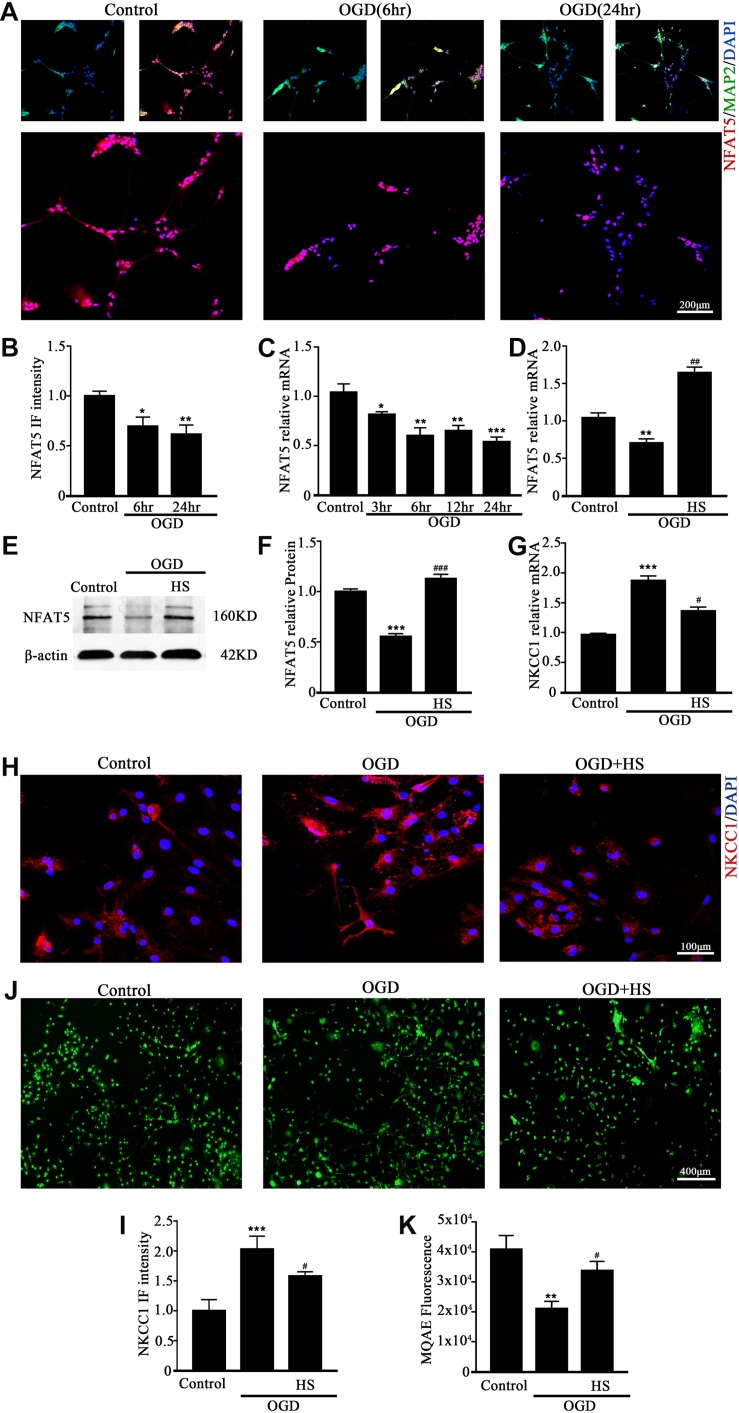
HS treatment inhibited NKCC1 expression. **(A)** Confocal images showing expression of NFAT5 in neurons. **(B)** The intensity of NFAT5 fluorescence was determined in 10 fields/well and divided by the number of cells counterstained with DAPI. **(C)** NFAT5 mRNA expression level at 3, 6, 12, and 24 h after OGD. **(D)** Graphical representation of the fold changes of NFAT5. HS (100 mM) treatment increased NFAT5 expression compared to OGD. **(E)** The protein expression level of NFAT5 examined in Control, OGD and HS groups. **(F)** Quantification of independent blots. **(G)** NKCC1 mRNA expression level after treatment with HS. **(H)** Confocal image demonstrating NKCC1 expression in neurons after treatment with HS (100 mM). **(I)** The intensity of NKCC1 was determined in 10 fields/well and divided by the number of cells counterstained with DAPI. **(J,K)** Fluorescence imaging of Cl^–^ via MQAE staining in neurons. The green fluorescence indicates the intensity of MQAE. The values represent the mean ± SEM. ^∗^*p* < 0.05, ^∗∗^*p* < 0.01, ^∗∗∗^*p* < 0.001 versus control (Tukey’s test after one-way ANOVA). ^#^*p* < 0.05, ^##^*p* < 0.01, ^###^*p* < 0.001versus OGD (Tukey’s test after one-way ANOVA).

**FIGURE 4:**
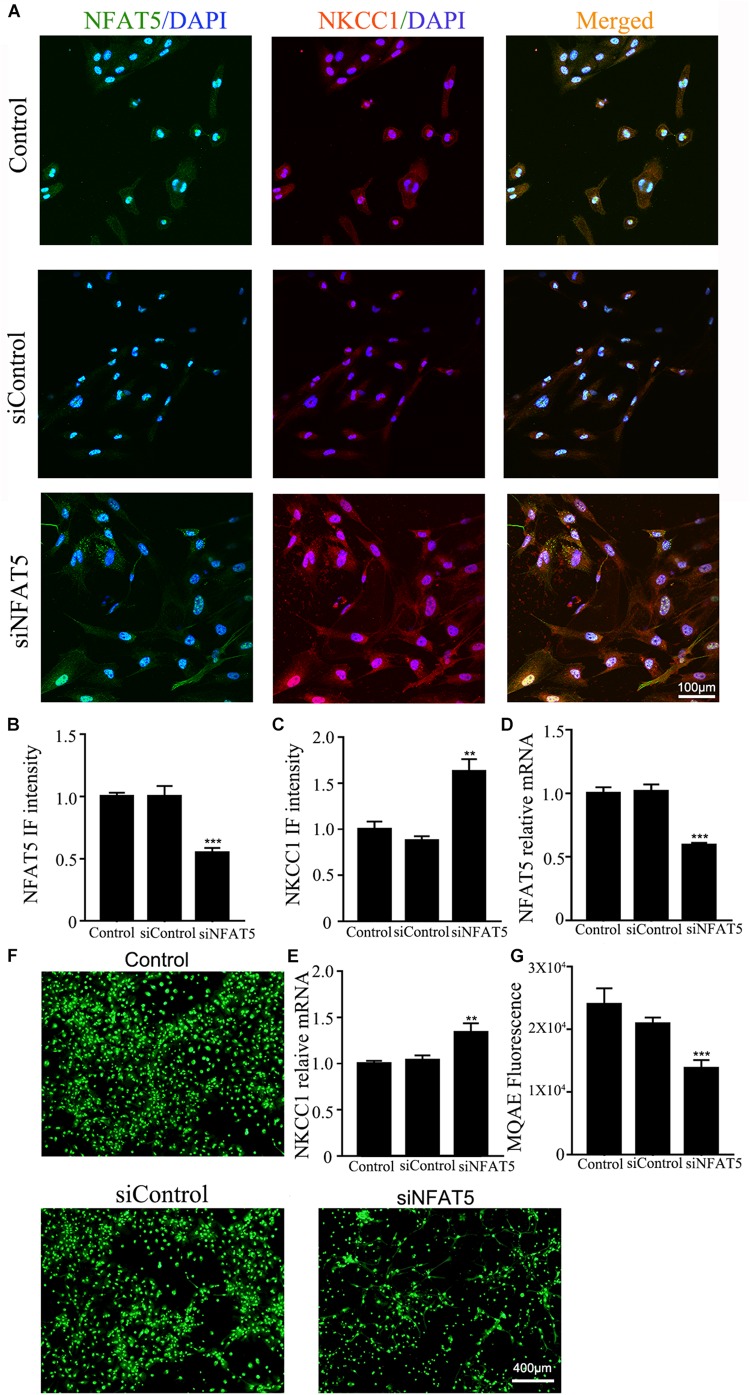
NAFT5 knock down upregulated NKCC1 expression. **(A)** Confocal image demonstrating NKCC1 expression in neurons after transfection with NFAT5-specific siRNA. **(B,C)** The intensity of NFAT5 and NKCC1 fluorescence were determined in 10 fields/well and divided by the number of cells counterstained with DAPI. **(D,E)** NFAT5 and NKCC1 mRNA expression level in neurons at 36hr after transfection. **(F,G)** Fluorescence imaging of Cl^–^ via MQAE staining in neurons. The green fluorescence indicates the fluorescence intensity of MQAE. The values represent the mean ± SEM. ^∗∗^*p* < 0.01, ^∗∗∗^*p* < 0.001 versus control (Tukey’s test after one-way ANOVA).

NFAT5 mRNA was downregulated after neonatal HI (Supplementary Figure S2A). Western blotting (Supplementary Figures S2B,C) and immunohistochemistry (Supplementary Figures S2D,E) were used to confirm NFAT5 expression in coronal brain slices following neonatal HI. NFAT5 mRNA expression and NFAT5 labeling cells in hippocampal cells was reduced compared to in the sham group after neonatal HI. *In vitro*, NFAT5 fluorescence intensity was reduced at 6hr and 24hr after OGD (Figures 3A,B and Supplementary Figure S1A). NFAT5 mRNA was downregulated at 3, 6, 12, and 24 h (Figure 3C). Next, we used HS to reverse the OGD-induced downregulation of NFAT5. HS treatment stimulated NFAT5 expression in neurons, as demonstrated by western blotting (Figures 3E,F) and q-PCR (Figure 3D) after OGD. HS treatment also reduced NKCC1 expression (Figures 3G–I) and neuronal [Cl^–^]_*i*_ (Figures 3J,K) after OGD. To further confirm the role of NFAT5 in NKCC1 expression, NFAT5-specific siRNA (Supplementary Table S2 and Supplementary Figure S3) was used to simulate OGD-induced downregulation of NFAT5 under physiological conditions. Transfection with NFAT5-specific siRNA effectively reduced NFAT5 fluorescence intensity (Figures 4A,B) and mRNA expression level (Figure 4D). NKCC1 fluorescence intensity (Figures 4A,C), mRNA expression level (Figure 4E) and [Cl^–^]_*i*_ (Figures 4F,G) were significantly upregulated in NFAT5-specific siRNA-transfected hippocampal neurons. These results suggest that OGD-induced NFAT5 downregulation was vital for aberrant NKCC1 expression in hippocampal neurons.

### NFAT5 and HIF-1α Coordinate Expression of NKCC1 in Hippocampal Neurons

**FIGURE 5:**
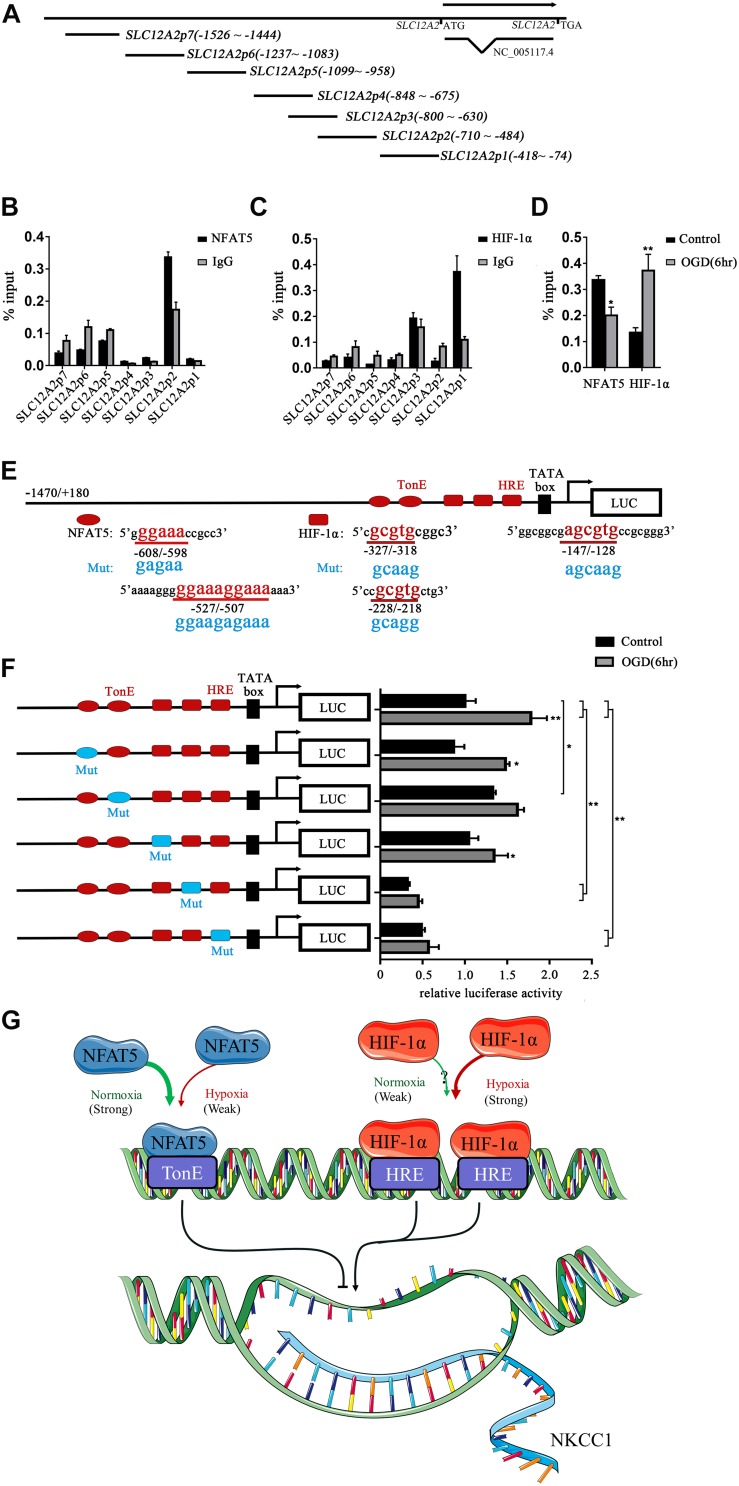
NFAT5 and HIF-1α transcriptional regulation of NKCC1. **(A)** Illustration of the primer sets at the NKCC1 promoter used in the ChIP assays. **(B,C)** Enrichment of NFAT5 and HIF-1α at the NKCC1 promoter region was determined by the primers shown in Supplementary Table S1. ChIP-qPCR data were normalized by the percent input method. **(D)** ChIP assays of NFAT5 and HIF-1α in the presence or absence of OGD. Hippocampal neurons were collected for ChIP assay at 6 h after OGD. ChIP-qPCR data were normalized using the fold enrichment method (ChIP signals were divided by IgG signals). **(E)** Prediction of NFAT5- and HIF-1α-binding sites in the NKCC1 promoter and mutation sites of the luciferase expression vector. In the synthesis of the NKCC1 promoter, point mutations were designed at two binding sites of NFAT5 and three binding sites of HIF-1α. **(F)** Effect of NFAT5 and HIF-1α on the transcriptional activity of NKCC1 promoters with different point mutations. In the synthesis of the NKCC1 promoter, point mutations were designed at the NFAT5- and HIF-1α-binding sites. The pGL3-basic plasmid containing the NKCC1 promoter and point-mutated promoters or empty-vector controls were transfected into PC12 cells. The OGD group was assessed for luciferase(activity at 6 h after OGD (at 36 h after transfection). **(G)** Cartoon indicating the relationship between NKCC1, NFAT5 and HIF-1α in hippocampal neurons. NKCC1 transcription is negatively regulated by NFAT5, while HIF-1α positively regulates its expression. The values represent the mean ± SEM (*n* = 5 per group). ^∗^*P* < 0.05, ^∗∗^*P* < 0.01.)

To determine whether NFAT5 and HIF-1α directly regulate NKCC1 expression, we examined the binding of NFAT5 and HIF-1α at NKCC1 promoter regions using ChIP assays. Seven pairs of primers (Supplementary Table S1) were designed at the NKCC1 promoter to determine the binding sites of NFAT5 and HIF-1α (Figure 5A). The purified DNA eluate was quantitated by qPCR, and the results are shown in Figures 5B,C. NFAT5 specifically bound at *SLC12A2p2* under normal conditions, while it had relatively weak binding activity after OGD (Figure 5D). However, HIF-1α was specifically highly concentrated at *SLC12A2p1* after OGD compared to in the control group (Figure 5D). These results demonstrate that NFAT5 and HIF-1α directly occupy different NKCC1 promoter regions.

Sequence analysis indicated the presence of two putative NFAT5 binding sites (TonE) at *SLC12A2p2* and three putative HIF-1α sites (HRE) at *SLC12A2p1* in the NKCC1 promoter. We designed an NKCC1-specific luciferin promoter (−1470/ + 180) using pGL3-basic, and point mutated pGL3-NKCC1p at TonEs and HREs respectively as shown in Figure 5E. Point mutating the second TonE increased control group luciferase activity compared to pGL3-NKCC1p, and separately point mutating the second and third HRE significantly reduced the luciferase activity of the control and OGD groups compared to pGL3-NKCC1p (Figure 5F). These results strongly suggest that NFAT5 binds to the second TonE at *SLC12A2p2* and inhibits NKCC1 promotor activity. HIF-1α is highly likely to bind the second HRE and third HRE at *SLC12A2p1* and increases NKCC1 promotor activity (Figure 5G).

### Relationship Between NFAT5 and HIF-1α in Hippocampal Neurons After OGD

**FIGURE 6:**
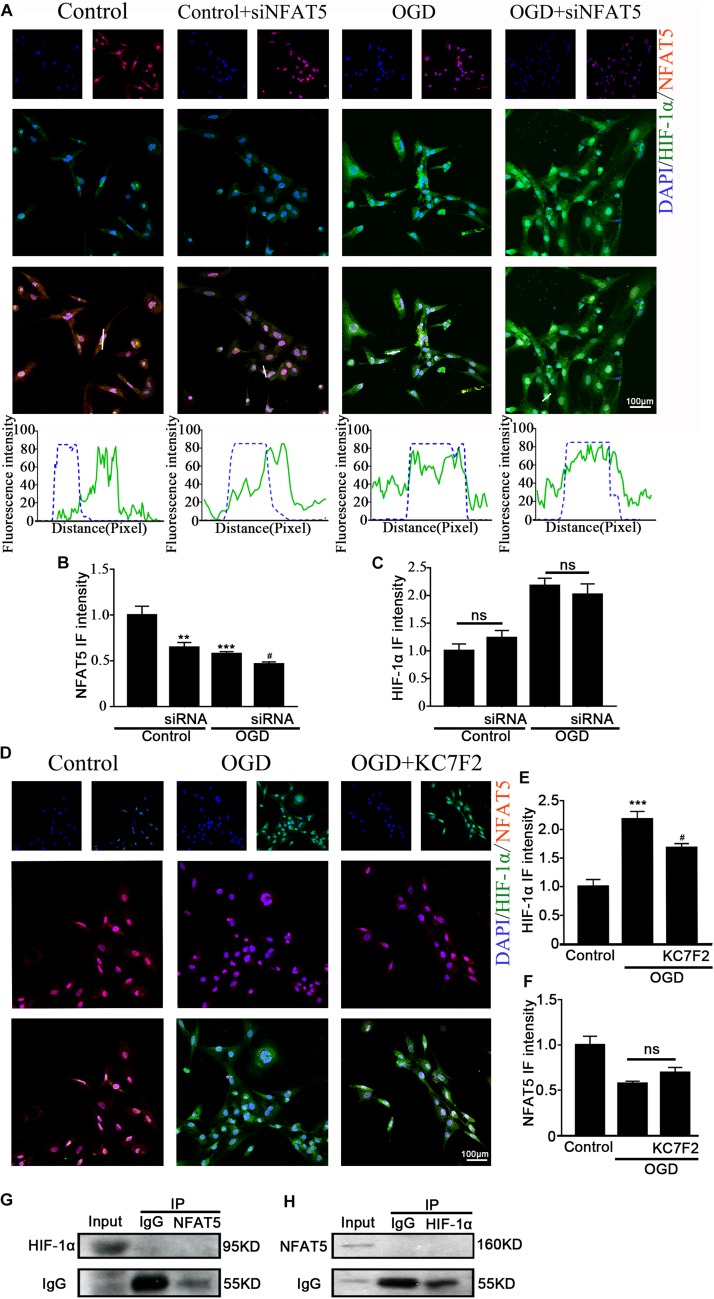
Interaction between NFAT5 and HIF-1α after OGD. (**A**, top) Confocal image demonstrating NKCC1 expression in neurons after transfection with NFAT5-specific siRNA. (**A**, bottom) Side overlap of two peaks indicated as a partial co-localization (highlighted by the white line). **(B,C)** The intensity of NFAT5 and HIF-1α fluorescence was determined in 10 fields/well and divided by the number of cells counterstained with DAPI. **(D)** Confocal image demonstrating NFAT5 expression in neurons after treatment with KC7F2. **(E,F)** The intensity of NFAT5 and HIF-1α fluorescence was determined in 10 fields/well and divided by the number of cells counterstained with DAPI. **(G)** Co-IP of endogenous NFAT5 and HIF-1α in primary cultured hippocampal neurons assessed in neurons at 6hr after OGD. After immunoprecipitation with NFAT5 beads, the purified proteins, along with input samples, were detected by western blotting with anti-HIF-1α and anti-IgG antibodies. **(H)** After immunoprecipitation with HIF-1α beads, the purified proteins, along with input samples, were detected by western blotting with anti-NFAT5 and anti-IgG antibodies. The values represent the mean ± SEM. ^∗∗^*p* < 0.01, ^∗∗∗^*p* < 0.001 versus control (Tukey’s test after one-way ANOVA). ^#^*p* < 0.05 versus OGD (Tukey’s test after one-way ANOVA).

Previous studies showed that NFAT5 and HIF-1α worked in coordination in macrophages and nucleus pulposus cells (Gogate et al., 2012; Neubert et al., 2019). The above results suggested that they both regulate OGD-induced aberrant NKCC1 expression in hippocampal neurons. Next, we explored the relationship between NFAT5 and HIF-1α. Knocking down NFAT5 under normal conditions or after OGD did not influence HIF-1α expression or nuclear localization (Figures 6A–C and Supplementary Figure S4B). Chemically inhibiting HIF-1α pathway by treating neurons with KC7F2 also did not affect NFAT5 expression (Figures 6D–F and Supplementary Figure S4A). These results suggest that NFAT5 and HIF-1α do not regulate each other’s expression. Next, Co-IP was used to confirm whether or not NFAT5 and HIF-1α directly bind to each other. Hippocampal neurons were collected 6 h after OGD, and cell lysates was purified with protein A- or protein G-coupled Sepharose. The purified proteins, along with input samples, were detected by western blotting with anti-HIF-1α antibodies. Figure 6G shows that NFAT5 was not co-immunoprecipitated with HIF-1α. A reverse Co-IP experiment was also used to test the interaction. After immunoprecipitation with HIF-1α beads, the immunoprecipitated proteins, along with input samples, were detected by western blotting with anti-NFAT5 antibodies. Figure 6H shows that HIF-1α was not co-immunoprecipitated with NFAT5 either. It seems that there is no mutual regulation between NFAT5 and HIF-1α in hippocampal neurons after OGD.

## Discussion

Upregulation of the NKCC1 chloride importer is observed in a wide range of conditions including neonatal and adult epilepsy, autism spectrum disorder, chronic pain, spinal cord lesions, brain trauma, cerebral edema, stress, neurogenic hypertension, cerebral artery occlusion, diabetic ketoacidosis and HI brain damage (Ben-Ari, 2017; Luo et al., 2018). Our previous research showed that inhibition of NKCC1 expression reduced neonatal HI-induced seizure susceptibility via lessening aberrant hippocampal neurogenesis and BBB leakage by rescuing the expression of tight junction-related protein zona occludens-1 (ZO-1) in endothelial cells (Hu et al., 2017; Luo et al., 2018). The consequences of aberrant NKCC1 expression has been well demonstrated, but the potential mechanism has barely been explored in HIE. In this study, we revealed that hypoxia induced NKCC1 upregulation was dependent on both NFAT5 and HIF-1α activity. Direct HIF-1α binding to HREs in the NKCC1 promoter at −228/−218 and −147/−128 positively regulates NKCC1 expression, while direct NFAT5 binding to TonE in the NKCC1 promoter at −527/−507 negatively regulates NKCC1 expression in hippocampal neurons. The cooperation between NFAT5 and HIF-1α is critical for the proper expression of NKCC1 in hippocampal neurons.

NKCC1 was distinctly upregulated *in vivo* (hippocampus brain slices) and *in vitro* (hippocampal neurons). NKCC1 mRNA peaking time point was early than the NKCC1 protein level in SD rats after neonatal HI. In primary cultured neurons, NKCC1 mRNA peaked at about 6hr after OGD, while the protein level was continuously increased after OGD up to 24 h, which is when it peaked. The changes *in vivo* and *in vitro* were not completely consistent, which may be because NKCC1 is expressed in glia cells too and its expression was increased under hypoxic conditions (Chen and Sun, 2005; Jayakumar and Norenberg, 2010; Xu et al., 2017).

Although aberrant NKCC1 expression in HIE has been demonstrated (Hu et al., 2017; Luo et al., 2018), the underlying mechanism is unclear. Previous studies showed that HIF-1α mediates NKCC1 expression in epithelial cells during hypoxia, and traumatic brain injury (TBI)-induced NKCC1 upregulation in turn promotes HIF-1α activity in the hippocampus (Ibla et al., 2006; Lu et al., 2015). The impact of HIF on neuronal survival is controversial (Vangeison et al., 2008; Sheldon et al., 2009; Barteczek et al., 2017; Soni and Padwad, 2017). On the one hand HIF target genes include erythropoietin and vascular endothelial growth factor, both of which are neuroprotective and result in reduced infarct size after cerebral ischemia (Ratan et al., 2004). On the other hand, HIF induces the mRNA expression of pro-death genes such as *Bcl-2* and adenovirus E1B 19 kDa-interacting protein 3 (*BNIP3)* and inhibits heat shock protein 70 (*Hsp* 70) mRNA expression (Gogate et al., 2012). To characterize the role of HIF-1α in NKCC1 expression, we checked the expression of HIF-1α in hippocampal neurons after OGD. HIF-1α expression and nuclear localization increased at 6hr and decreased to normal levels at 24 h, which was consistent with the NKCC1 mRNA expression changes. Chemically inhibiting the HIF-1α transcription activation significantly ameliorated OGD-induced aberrant NKCC1 expression and reduced neuronal [Cl^–^]_*i*_. The ChIP assay and site-directed mutagenesis provided further mechanistic details on the possible positive regulatory role of HIF-1α. HIF-1α is enriched at and directly binds to the NKCC1 promoter region after OGD, and point mutations in the second HRE and third HRE at *SLC12A2p1* (Figures 5A,E) inhibited OGD-induced NKCC1 promoter activity suggesting that direct HIF-1αbinding to HREs in the NKCC1 promoter positively regulates NKCC1 expression in hippocampal neurons.

HS can be used to treat brain edema in clinical settings, which leads to inhibition of NKCC1 expression (Deng et al., 2016; Wu et al., 2019). HS also significantly stimulates NFAT5 expression, which is highly expressed in the fetal brain, and genetically knocking out NFAT5 resulted in an edematous stillborn fetus (Yang et al., 2018). NFAT5 was recently characterized as a hypoxia-inducible protein, but the changes of NFAT5 expression after hypoxia is controversial (Hao et al., 2014; Dobierzewska et al., 2015; Xia et al., 2017). To characterize the role of NFAT5 in NKCC1 expression, we first checked the expression of NFAT5 *in vivo* and *in vitro* under hypoxic conditions. NFAT5 expression decreased after hypoxia. HS treatment reversed the OGD-induced downregulation of NFAT5, which significantly hampered OGD-induced NKCC1 expression and neuronal [Cl^–^]_*i*_. HS treatment does not specifically affect NFAT5 expression, so we used NFAT5-specific siRNA to simulate OGD-induced NFAT5 downregulation. Consistent with our expectations, NKCC1 expression and [Cl^–^]_*i*_ were increased in hippocampal neurons after NFAT5 knock down. ChIP assay and site-directed mutagenesis provided further mechanistic details on the possible role of NFAT5 in NKCC1 expression. NFAT5 is enriched and directly binds to the NKCC1 promoter region after OGD and point mutations in the second TonE at *SLC12A2p2* (Figures 5A,E) increased background NKCC1 promoter activity, suggesting that direct NFAT5 binding to TonE in the NKCC1 promoter negatively regulates NKCC1 expression in hippocampal neurons.

Previous research demonstrated that HIF-1α and NFAT5 regulated diet-independent Na^+^ accumulation-induced pro-inflammatory activation of mouse macrophages, and NFAT5 was important for Na^+^ accumulation-induced HIF-1α upregulation (Neubert et al., 2019). NFAT5 stimulated HSP90 expression while HIF-1α inhibited HSP90 expression in hypoxic nucleus pulposus cells (Woo et al., 2002; Gogate et al., 2012). In hippocampal neurons, it is largely unknown how the NFAT5 and HIF-1α-dependent pathways interact. We provide evidence that they cooperatively regulate OGD-induced aberrant NKCC1 expression via interaction with their corresponding binding sites. However, mutual regulation between NFAT5 and HIF-1α was not observed after hypoxia. It seems that they are like colleagues that work together but never communicate under hypoxic conditions. Does the up-stream of HIF-1 and NFAT5 crossed under hypoxia condition is not clear yet, and it is worth paying more effort on.

In this study, we revealed the potential mechanism of aberrant NKCC1 expression in hippocampal neurons in HIE. The two transcription factors, HIF-1α and NFAT5, are critical for aberrant NKCC1 expression. HIF-1α is upregulated after OGD and directly binds to the NKCC1 promotor, which positively regulates NKCC1 expression after OGD. NFAT5 is downregulated after OGD and also directly binds to the NKCC1 promotor, which negatively regulates NKCC1 expression. The cooperation of HIF-1α and NFAT5 in the regulation of NKCC1 expression is a potential therapeutic target for HIE.

## Data Availability Statement

All datasets generated for this study are included in the article/Supplementary Material.

## Ethics Statement

The animal study was reviewed and approved by the Care and Use Committee of Wuhan University Medical School.

## Author Contributions

X-LY and B-WP conceived and designed the experiments. X-LY, M-LZ, LS, and G-TJ performed the experiments. X-LY, G-TJ, J-JC, M-LZ, and LS analyzed the data. SH, JY, W-HL, X-HH, and B-WP contributed to the reagents, materials, and analysis tools. X-LY and B-WP wrote the manuscript. All authors reviewed and approved the final manuscript.

## Conflict of Interest

The authors declare that the research was conducted in the absence of any commercial or financial relationships that could be construed as a potential conflict of interest.

## Footnotes

1 https://imagej.nih.gov/ij/
