# Supplementary material for: NFAT5 and HIF-1α Coordinate to Regulate NKCC1 Expression in Hippocampal Neurons After Hypoxia-Ischemia
Source: Front Cell Dev Biol. 2019 Dec 13;7:339. doi: 10.3389/fcell.2019.00339 (PMC6923656; doi:10.3389/fcell.2019.00339)
Supplement: Supplementary file 1 [file Data_Sheet_1.docx]

**SUPPLEMENTARY MATERIALS**

## Immunofluorescence

Rats in each group were deeply anesthetized with 10% chloral hydrate and transcardially perfused first with PBS and then fixed in 4% paraformaldehyde solution at room temperature, dehydrated, and embedded in paraffin at 24 h after HI. Post-fixation, the brains were removed and cryoprotected in 20% sucrose and 30% sucrose solutions and dehydrated by 30% sucrose for 72 h at 4 °C. Serial coronal sections (5-μm-thick with injury epicenter located centrally) prepared with cryotome (Leica, Wetzlar, Germany) was used for immunofluorescence labeling. Sections were incubated with a blocking solution (5% FBS) for 30 min at 37 °C. The tissue slices were then incubated overnight with antibodies. On the following day, the sections protected from light were washed and subsequently incubated with secondary antibodies Cy3-conjugated IgG (H+L) (1:100) for 2 h at 37 °C. Images were obtained using a confocal microscope (Leica-LCS-SP8-STED).

For the assessment of NKCC1, NFAT5, and HIF-1α expression in neurons, cells were fixed with methanol, washed with PBS-T, and incubated at 4 °C with anti-NKCC1, anti-NFAT5, anti-HIF-1α antibodies and IgG (Abcam, Cambridge, MA; used at 1:50). Subsequently, cells were washed with PBS-T before incubation with mixtures of secondary antibodies: Alexa Fluor^®^ 488 Conjugates (H +L) (1:100) and Cy3-conjugated IgG (H +L) (1:100) diluted in blocking buffer for 2 h in the dark at room temperature. The cells were washed three times in PBS-T before they were mounted using DAPI. Finally, cellular co-localization was captured using confocal microscope (Leica-LCS-SP8-STED).

## qPCR

The quantity of total RNA was measured by a UV spectrophotometer (Biochrom Ltd., UK). Next, reverse transcription was performed using a cDNA synthesis kit (TaKaRa Biotechnology). QPCR was performed on a SYBR-Green premix (Trans Gen Biotech) according to the manufacturer’s specification with the primers (Sangon Biotech, Shanghai, Co., Ltd.). All primers are listed in Table 1.The cycling parameters for the CFX96 sequence detection system were 95 °C for 5 min; 40 cycles of 95 °C for 15 s, 58 °C for 30 s, and 72 °C for 20 s. The expression of target genes was normalized to the mRNA level of β-actin as an internal control. The ΔΔCt values of each group were analyzed, and the mRNA expression of different groups was normalized to 2^−ΔΔCt^.

## Western blotting

Western blotting was performed according to the manufacturer’s specification. Neurons were collected after OGD. Proteins were extracted by homogenizing in RIPA buffer (Santa Cruz Biotechnology, Santa Cruz, CA) with PMSF, and further centrifuged at 12,000 rpm at 4 °C for 10 min. The protein concentrations were measured using a detergent compatible assay (Bio-Rad, Dc protein assay). Equal amounts of protein were loaded on an SDS-PAGE gel. After electrophoresis and transfer to a polyvinylidene fluoride (PVDF) membrane, the membranes were blocked by 5% skimmed milk for 3 hr. The membranes were incubated with the HIF-1α (Abcam, Cambridge, MA; used at 1:1000), NFAT5 (Abcam, Cambridge, MA; used at 1:500) antibodies overnight at 4 °C. After incubation, the membranes were washed at least three times with TBST (TBS containing 0.2% Tween-20) and were then incubated for 1.5 hr with secondary antibodies at room temperature. The membranes were washed again with TBST three times. Finally, the reaction was developed using a chemiluminescent reagent (ECL; Ecl Advantage Inc., Menlo Park, California, USA) and the bands of different proteins were detected using an imaging densitometer (Bio-Rad, Foster City, CA, USA). The data were analyzed using ImageJ software (version 1.41).

**Table 1** Primers for PCR amplification and analysis

| **Primer name** | **Forward primer 5′-3′** | **Reverse primer 5′-3′** |
| --- | --- | --- |
| NKCC1*(SLC12A2)* | AGACTTCAACTCAGCCACTGT | CAAGGTCAAACCTCCATCATCA |
| *Nfat5* | AGCAGCTGGTGCTTTGAGTG | GCCAGTCGTTTTCATTGCTTTC |
| *Hif-1α* | CCACAGGACAGTACAGGATG | TCAAGTCGTGCTGAATAATACC |
| NKCC1-P | CCGCTCGAGATTAATATTGGGAAAGTTCAAGGTGGGGA | CCCAAGCTTTGGCAGAGGTTTGCCCAGCCCATC |
| *SLC12A2P1* | CGCCCTAAGGGAAACC | CGGACCCTGTGGACTC |
| *SLC12A2P2* | GCCCAACAGGAAAGCC | CTGTCGCGGATGCTCT |
| *SLC12A2P3* | GCACGGAGCGGTAGAG | GGGCGGTTTCCCTTAG |
| *SLC12A2P4* | GGCATCGCGGCTGGAAT | TGGCTGCGGGTGATGCT |
| *SLC12A2P5* | CAGTGTTAGGAAACGCAGT | AAACAAGCCCGCAGAG |
| *SLC12A2P6* | TAGAGTGAACGCAGGCATAA | GCTCTGCGGGCTTGTT |
| *SLC12A2P7* | CCTGGCTTCTCCTGATT | GCCAAGGCTTCTAATACAC |

**Table 2** sequences of siRNA

| **siRNA name** | **Forward primer 5′-3′** | **Reverse primer 5′-3′** |
| --- | --- | --- |
| **siControl (NC)** | GTTCTCCGAACGTGTCACGT | ACGTGACACGTTCGGAGAATT |
| **siNFAT5 (875)** | TTCTCCGAACGTGTCACGT | ACGTGACACGTTCGGAGAA |
| siNFAT5-1551 | GCACCTCACTATGTGCTTTCT | AGAAAGCACATAGTGAGGTGC |
| siNFAT5-1712 | GGTACAGCCTGAAACCCAACA | TGTTGGGTTTCAGGCTGTACC |
| siNFAT5-4178 | GCTGCAGCCTTCCATGTTTCA | TGAAACATGGAAGGCTGCAGC |


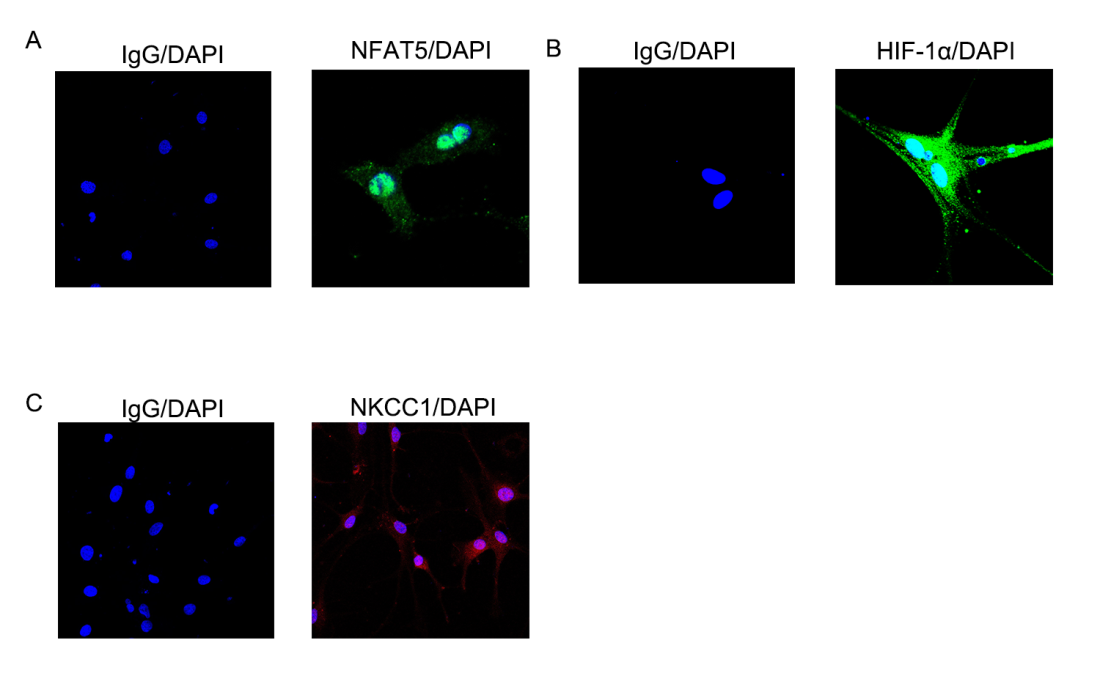


**Supplementary Fig. 1 IgG control for Immunofluorescence staining. (A) IgG control for NFAT5 staining in primary cultured hippocampal neurons. (B) IgG control for HIF-1α staining in primary cultured hippocampal neurons. (C) IgG control for NKCC1 staining in primary cultured hippocampal neurons.**


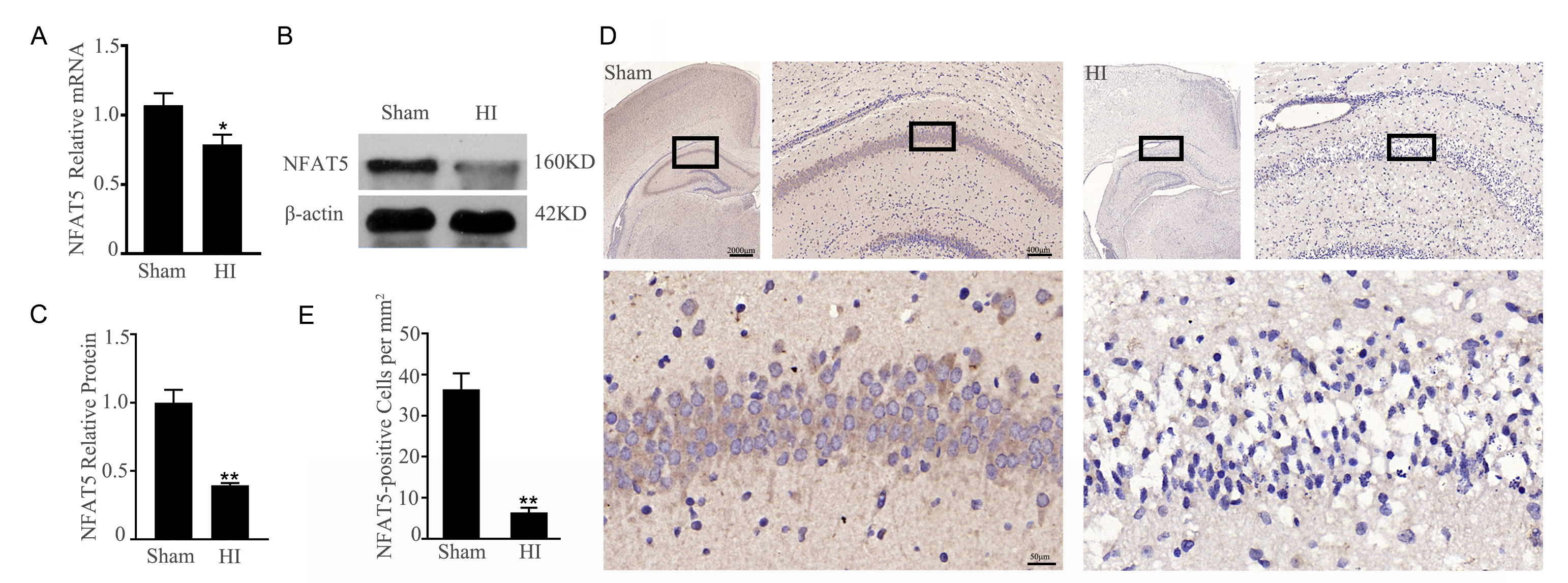


**Supplementary Fig. 2 NFAT5 is downregulated in hippocampus after neonatal HI. (**A) The NKCC1 mRNA expression level 6hr after neonatal HI. (B) The protein expression level of NFAT5 examined in Sham and HI groups. (C) Quantification of independent blots. (D,E) Ipsilateral hemisphere hippocampus from the HI and Sham groups were subjected to immunohistochemistry using an anti-NFAT5 antibody, and the number of NFAT5-positive cells per mm^2^ was counted. The values represent the mean ± SEM. ^*^*p* < 0.05, ^**^*p* < 0.01 versus Sham (Student’s t test).


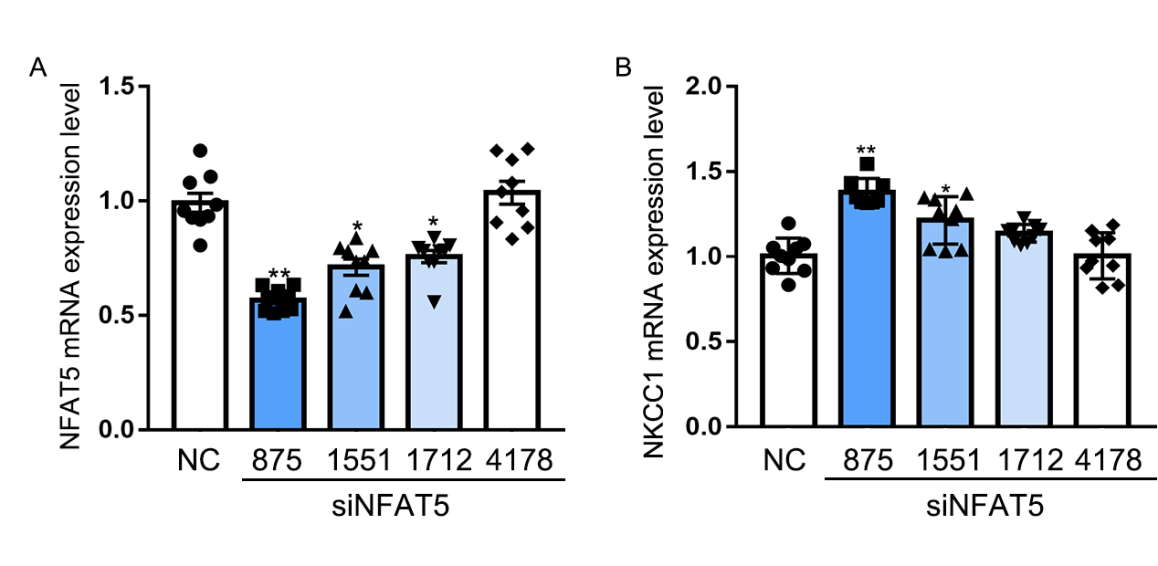


**Supplementary Fig. 3 NFAT5 and NKCC1 mRNA expression level in hippocampal neurons.** **(**A) The NFAT5 mRNA expression level in hippocampal neurons 36hr after transfected with potential NFAT5 siRNA respectively. The numbers represent siRNA binding site (bp). (B) The NKCC1 mRNA expression level in hippocampal neurons 36hr after transfected with potential NFAT5 siRNA respectively. The values represent the mean ± SEM. *P<0.05, **P<0.01 versus NC (Tukey’s test after one-way ANOVA).


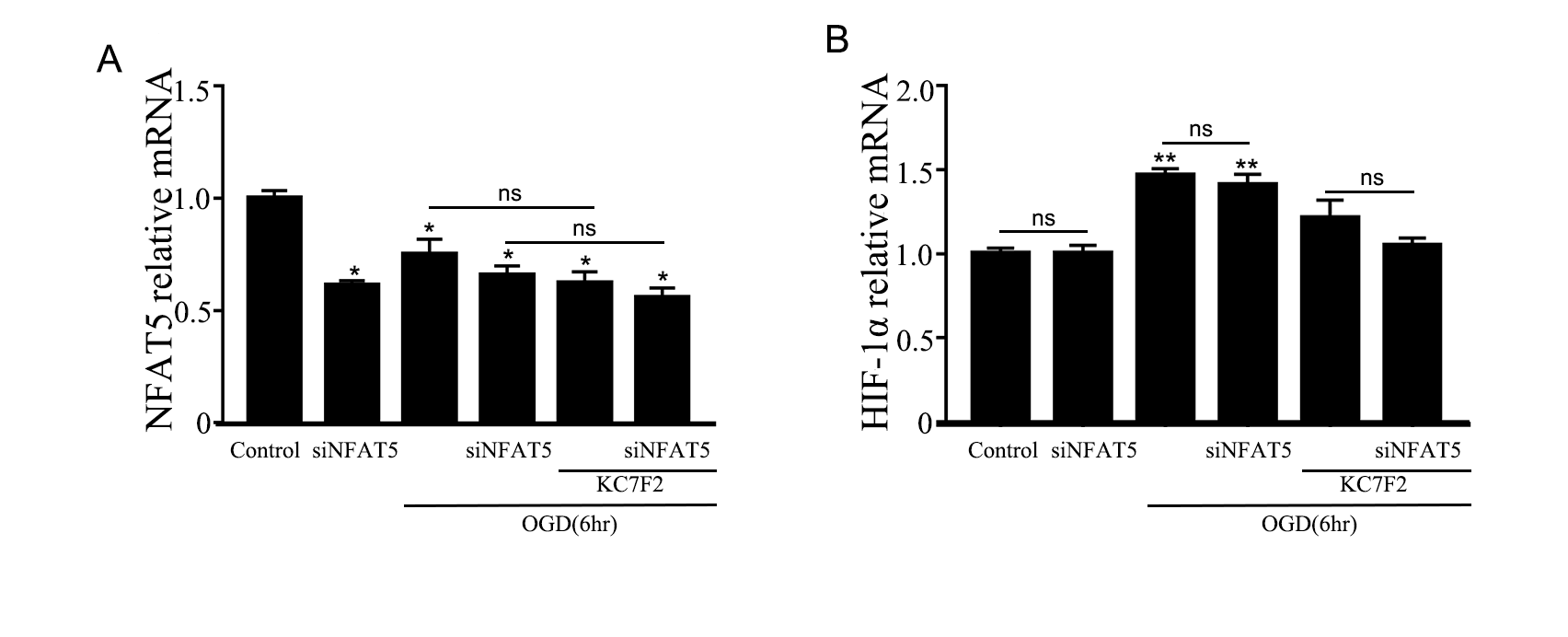


**Supplementary Fig. 4 NFAT5 and HIF-1α mRNA expression level in hippocampal neurons. (**A) The NFAT5 mRNA expression level in OGD group, siNFAT5 group and KC7F2 group fold to Control group. (B) The HIF-1α mRNA expression level in OGD group, siNFAT5 group and KC7F2 group fold to Control group. The values represent the mean ± SEM. ^*^*p* < 0.05, ^**^*p* < 0.01 versus control (Tukey’s test after one-way ANOVA).
